# Height Resolution of Antibody Spots Measured by Spinning-Disk Interferometry on the BioCD

**DOI:** 10.3390/mi7020031

**Published:** 2016-02-17

**Authors:** Kevin O’Brien, Ming Zhao, David Nolte

**Affiliations:** 1 Department of Physics, University of California Berkeley, Berkeley, CA 94720-7300, USA; kpobrien@berkeley.edu; 2 Department of Physics, Purdue University, West Lafayette, IN 47907, USA; mingzhao03@gmail.com

**Keywords:** differential phase contrast, common-path interferometry, silicon metrology, molecular interferometry, protein immobilization

## Abstract

Spinning-disc interferometry (SDI) is a high-speed laser scanning approach to surface metrology that uses common-path interferometry to measure protein spots on a BioCD disk. The measurement sensitivity depends on the scanning pitch and on the time-base. Based on high-resolution laser scanning images of printed antibody spots, we quantify the protein sensitivity as a function of the scan parameters. For smoothly printed antibody spots scanned with a transverse spatial resolution of 1 μm, the surface height precision for a single 100 μm diameter protein spot is approximately 1 pm. This detection sensitivity sets the fundamental limit of detection for label-free BioCD biosensors performing immunoassays.

## 1. Introduction to Spinning-Disc interferometry

The BioCD [1] is a spinning-disc detection platform that uses laser interferometry to detect protein bound on the disc surface [2]. The interferometric detection uses common path configurations that are insensitive to surface vibrations [3]. The common path configurations include microdiffraction [1], adaptive optical [4], phase contrast [5] and in-line optical configurations [6]. In-line interferometric phase quadrature for sensitive phase-to-intensity transduction is achieved using an eighth-wave thermal oxide on silicon wafers. Direct imaging of protein on the wafers is possible, called molecular interferometric imaging (MI2), and has the advantage of high spatial resolution for the study of protein binding on printed antibody arrays [7,8,9]. However, MI2 is not practical for the imaging of large areas. The entire disk surface can be measured rapidly with high speed using point laser detection on a spinning wafer. Immunoassays with high throughput have been performed using the spinning format for prostate specific antigen detection [10,11] with assay sensitivity in human serum samples down to 4 ng/mL.

High rotation rates (6000 rpm) of the BioCD enable high-speed detection of large numbers of protein spots on a single silicon wafer. For instance, a BioCD containing 50,000 printed antibody spots can be scanned in 4 min. However, there are tradeoffs between throughput and surface height sensitivity related to the size of the probe laser beam and the pitch between successive scan radii. This paper explores these trade-offs and establishes the metrology limit for spinning-disc interferometry. The metrology sensitivity of spinning disk interferometry of printed protein surfaces sets the low bound for surface-height sensitivity. The metrology limit relates to changes in the surface constituents during the spinning scan itself. For instance, altered surface sensitivity was observed for changing water adsorption on the protein layers [12]. In this paper, the fundamental metrology limits of spinning-disk interferometry on the BioCD are established to be approximately 1 pm per 100 μm diameter antibody spot.

## 2. Common-Path Interferometry

**Figure 1:**
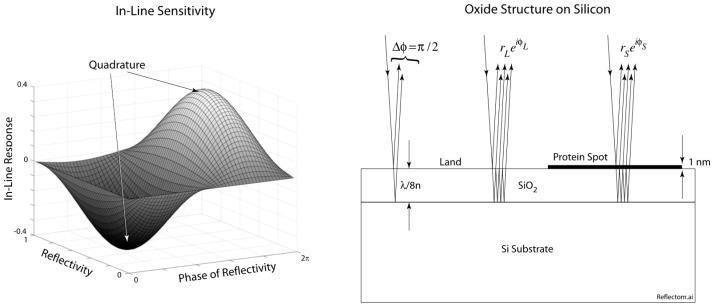
In-line surface phase quadrature is established with an eighth-wave layer of SiO_2_ on silicon. Immobilized protein on the surface converts the optical phase to intensity modulation when the surface reflectance equals ±0.577*i*.

Reflectance spectroscopy is a common detection approach to detect bound proteins, and several colorimetric biosensors have been based on this principle [13,14,15,16,17]. The BioCD with inline quadrature is based on the principle of phase quadrature for maximum phase detection sensitivity. Quadrature is achieved with an eighth-wavelength thickness of thermal oxide on silicon, shown in Figure 1. The partial wave reflected from the top surface has a π/2 phase relative to the partial wave reflected from the bottom of the oxide layer. Double-pass through the eighth-wave layer creates an optical path length difference of a quarter wave for a π/2 phase. A single detection wavelength can be chosen near the optimum, and the surface can be observed either by scanning an in-line BioCD [3,11,18], or observed in an imaging system [7,9,19].

For a surface with an original reflection coefficient *r*, applying a thin protein layer changes the reflection to r' [3].

(1) $$r'=r+\left[\frac{\left(r_{p}-r)(1-rr_{p}\right)}{\left(1-{r_{p}}^{2}\right)}+r\frac{\tan\theta_{p}}{\tan\theta_{0}}\right]\frac{4i\pi dn_{p}\cos\theta_{p}}{\lambda }$$

where *d* is the thickness of the protein, λ is the probe wavelength, $\theta_{0}$ is the incidence angle, $n_{p}$ is the refractive index of the protein layer, and

(2) $$r_{p}=\frac{\sin(\theta_{p}-\theta_{0})}{\sin(\theta_{p}+\theta_{0})}\;\mathrm{and}\;n_{p}\sin\theta_{p}=\sin\theta_{0}$$

Equation (1) simplifies at normal incidence to

(3) $$r'=r+\frac{{\left(1+r\right)}^{2}}{\cos\theta_{0}}\frac{\pi i}{\lambda }(1-{n_{p}}^{2})d$$

where $n_{p}$ and *d* are the refractive index and the thickness of the protein layer. The detector output current is proportional to the quantity

(4) iIL(x)=−2ϕIm|r|2[g2(x)⊗h(x)]iDPC(x)=−2ϕRe|r|2[(d(x)⋅g(x)⊗h(x)]

where d(x) is Dawson function (Hilbert transformation of the gaussian function g(x)), convolution is signified by the symbol ⊗, and the phase functions are

(5) ϕRe=4πnpcosθ0λRe((rp−r)(1−rrp)r(1−rp2)+tanθptanθ0)ϕIm=4πnpcosθ0λIm((rp−r)(1−rrp)r(1−rp2))

The sensitivity function φ_Im_ for in-line detection is plotted on the right of Figure 1, showing the quadrature condition. Therefore, optical phase is directly transduced to intensity, and increasing protein loads cause increasing intensity modulation as the protein spots on the disc pass through the detection laser beam.

## 3. Optical System and Characterization

**Figure 2:**
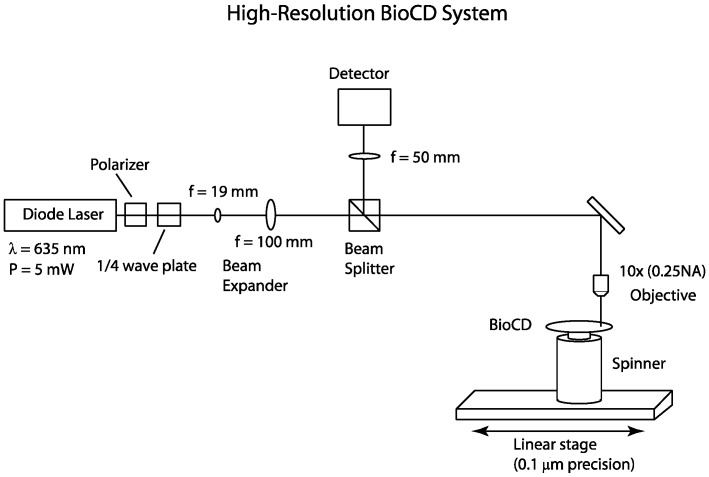
Optical layout for BioCD scanning. The diode laser operates at a wavelength of 635 nm. The disc is positioned on a linear translation stage with 1 μm precision for radial scanning. The beam expander controls the beam size at the disc.

The optical setup for the 1 μm resolution spinning disk interferometer [20] is shown in Figure 2. The light source is a 5 mW 635 nm diode laser (Model 31-0144-000, Coherent Inc, Santa Clara, CA, USA) with the polarization controlled using a Glan-Thompson polarizer (Model 5525, New Focus, San Jose, CA, USA) and a Soleil Babinet compensator (Model 25215, Oriel Instruments, Irvine, CA, USA) configured as a quarter-wave plate. This setup produces circularly polarized light at the surface of the disk. The use of 635 nm wavelength, when shorter wavelengths would have higher resolution, is based on lower scattering by small dust or debris. The beam is expanded and focused by a 10× (0.25 NA) objective lens onto the BioCD, which is mounted on a spinner (Lincoln Laser Inc., Pheonix, AZ, USA). The spinner rotates the disk at 1200 rpm. The reflected light is captured by the objective lens and separated by the non-polarizing beam splitter (Model 02BC17MB.1, Newport, Irvine, CA, USA). The optical signal is converted to an electrical signal by a 125 MHz silicon PIN detector (Model 1801, New Focus, Irvine, CA, USA) and detected with an oscilloscope (Model TDS 540, Tektronix, Beaverton, OR, USA). The data are then transferred to a computer. A 50 mm lens is placed in front of the detector to focus the reflected light onto the 0.8 mm diameter active area. The sample is translated using a 0.1 μm precision linear stage (Model UTM100PP.1, Newport, Irvine, CA, USA) operated by a motion controller(Model ESP300 Universal Motion Controller, Newport, Irvine, CA, USA).

To achieve multiple resolutions and beam diameters, the beam expander is removed and appropriate lenses are used in place of the 10× objective. A 5× objective lens (0.12 NA) produces a 5 μm beam waist and a 100 μm focal length lens produces a 10 μm beam waist. The beam radius was measured by observing an MD Class BioCD which consists of 20 μm wide gold spokes on a dielectric mirror. The gold spokes are a known structure from which the theoretical intensity traces are calculated using Fraunhofer diffraction. The protein spots of interest in this study are located approximately 35 mm from the center of the disk. The desired pitch is approximately equal to the minimum beam waist (1 μm) and one finds that a time base of 10 μs produces a sample interval of 0.2 μs which at 35 mm is a travel distance traveled of 0.9 μm.

The Model 1801 photodetector has a noise-equivalent power (NEP) of 3.3 pW·Hz^−1/2^ from 0–10 MHz and 30 pW·Hz^−1/2^ from 10–200 MHz. Therefore the integrated noise from 0–130 MHz is 328 nW_rms_ and with a conversion gain of 2.4 × 10^4^ V/W, the expected output noise voltage is 7.8 mV_rms_. The maximum bandwidth of the scope is 20 MHz and, from the NEP characteristics of the 1801 Detector, the integrated noise is 105 nW. The conversion gain is 2.4 × 10^4^ V/W for a predicted output noise voltage of 2.5 mV.

## 4. SDI Detection of IgG Antibody Spots

**Figure 3:**
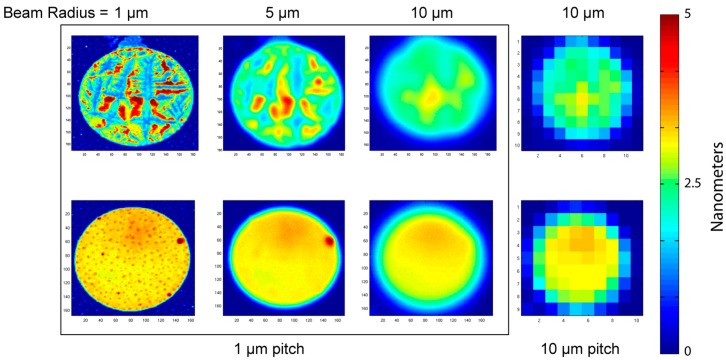
Two antibody spots with 100 μm diameters scanned with different resolutions and pitches. The six images on the left are with a 1 μm pitch at increasing laser beam diameters of 1, 5 and 10 μm. The two images on the right are with 10 μm pitch at 10 μm laser spot size.

Interferometric surface-protein detection is performed by spinning at high speed a BioCD carrying printed protein spots and interrogating the surface with a stationary focused laser spot. The size of the probe beam is a key factor that determines the appropriate pitch (distance between two scan tracks) and hence the time to read the disk. A comparison of spot sizes relative to the diameter of a printed antibody spot diameter is shown in Figure 3. The spots investigated were IgG antibodies printed on a diisocyanate chemically prepared surface. The IgG antibody is a common choice for many immunosorbent assays. For the scanning, a laser diameter of 2 μm has a pitch equal to 1 μm, for which the pitch is equal to the radius of the laser spot. This would require 100 tracks to scan a single printed protein spot with a diameter of 100 μm. Much more efficient scanning is possible with larger laser spot sizes, allowing larger pitches and fewer tracks per protein spot. However, the tradeoff is with resolution.

The resolution trend with increasing laser spot size is shown in Figure 3. The beam radius in the figure is 1, 5, and 10 μm (at 1 μm pitch), and a beam radius of 10 μm at a pitch of 10 μm pitch. Two printed antibody spots are sampled. One is characterized as “smooth”, but with water-spot residuals. The other is characterized as “rough” and displays tearing of the protein sheet and peeling away from the silicon wafer. The surface morphology of piezoelectric ink-jet printed protein is highly dependent on the surface chemistry. For well-immobilized prints, the antibody spot has a mean thickness of approximately 3 nm with a standard deviation of approximately 10%. However, when the immobilization chemistry is non-ideal, then the antibody protein layer can pull away from the surface, producing ragged features and large surface height deviations. A single BioCD has 768 printed antibody spots, and the morphology across the disc can vary, with large numbers of well-printed spots, but some number of faulty spots. Therefore, in this paper we picked one well-immobilized antibody spot, and one “worst-case” spot with ragged features. Increasing beam size averages over larger areas, suppressing the ragged features of the rough spot, and averaging over the water-spots of the smooth spot. The scan with 10 μm radius but 1 μm pitch is strongly oversampled, but provides high signal-to-noise and fidelity of the scan. The same laser spot size, but with a 10 μm pitch, also captures the average thickness of the antibody spot, but with much coarser sampling. Note that the time base was changed to match the change in pitch. There are considerably lower storage requirements for the coarse scan, and much faster scanning of full disks. A question this paper addresses is whether this large increase in speed and efficiency reduces measurement accuracy.

The scaling of surface height sensitivity for a single repeating scan on a single track provides the minimum possible surface height sensitivity for spinning-disk interferometry. The single track scaling experiment was performed by taking a number of single track data sets, then averaging and differencing them. For example, without averaging, the first two tracks were differenced and the standard deviation taken. For 1 average, tracks 1 and 2 were averaged, tracks 2 and 3 were averaged, then the two averages were differenced and the standard deviation taken. For *N* averages, tracks 1 to *N* and (*N* + 1) to 2*N* were averaged, then the difference and standard deviation of those two averages were taken. The time base was chosen to make the distance between consecutive data points approximately 1 μm. No averaging was done on the scope. The data were taken in the middle of the smooth protein (antibody) spot used in the other studies. The experiment was repeated for all beam radii of 1, 5 and 10 μm.

**Figure 4:**
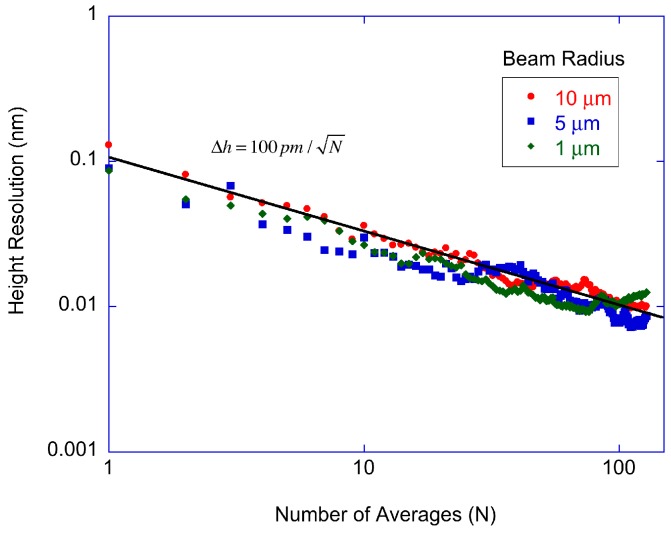
Single-track surface height resolution for repeated scans at the same radius. The data are for beam radii of 1, 5 and 10 μm. The height resolution for each pixel after 100 averages is 10 pm.

**Figure 5:**
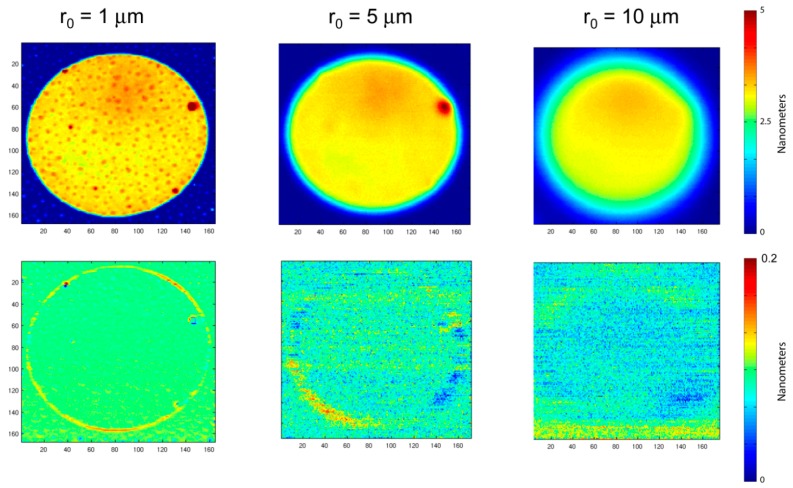
Top row: Scans at different beam radii. Color bar has a maximum at 5 nm. Bottom row: The difference of two consecutive scans (without dismount) showing edge effects and noise. Color bar on the bottom has a maximum at 0.2 nm.

The single-track averaging results are shown in Figure 4 as a function of the number of averages. By single-track is meant that the radius of the disc is not altered, allowing the laser to probe the same radial region multiple times. Keeping the radius fixed eliminates repositioning error along the radial direction. (Two-dimensional scans are shown later in Figure 5 in which the entire spot is measured by moving the disc position radially.) The number of averages is simply the number of rotations used in the average. The slight increase in noise above *N* = 100 averages is from 1/*f* drift of the measurement system, placing a practical limit on the number of averages. The shot noise limit for the detected power is shown as the solid line with a slope of −0.5 from the square-root dependence on averaging. The experimental data for the three beam spot sizes show the same square-root dependence, with no significant difference. Therefore, the protein height resolution for single-track scanning is limited by relative intensity noise or detector noise and is approximately a factor of 5 higher than the shot noise limit. For 100 averages, the protein height resolution is approximately 10 pm per pixel.

**Figure 6:**
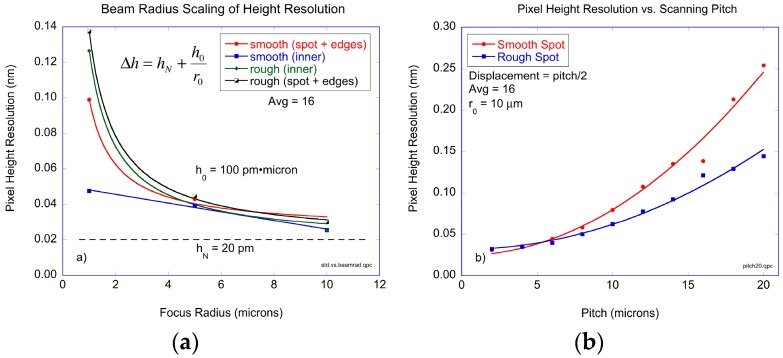
(**a**) 2D scan surface height resolution without dismount for the smooth and rough protein spots. (**b**) Pixel height noise as a function of the scan pitch for a fixed beam radius of 10 μm.

In assay operation, a BioCD is scanned over two dimensions by moving the radial tracks on a pitch that is equal to the spot radius. Therefore, a rescan of the same printed protein spot will have slight repositioning inaccuracy that will degrade the protein height resolution. Examples of the original 2D scans and the difference between two successive 2D scans are shown in Figure 5. The results of pixel height resolution in 2D scans is shown in Figure 6 as a function of the beam radius, all for a radial scan pitch equal to 1 μm. The two protein spots that were scanned were the smooth and rough spots described in Figure 3. In addition, the height resolutions were calculated considering only the inner regions (approximately 80% of the spot area centered on the middle of the spot to eliminate the edge pixels) of the printed antibody spot away from the edges, and then repeated including the edges. The data in Figure 6a are fit to a simple scaling relation:

(6) $$\Delta h=h_{N}+\frac{h_{0}}{r_{0}}$$

where *h_N_* is the noise floor, *r*_0_ is the beam radius, and the coefficient *h*_0_ sets the dependence on the beam radius. The rough antibody spot for both inner region plus outer edges, and the smooth spot when including the edges, scale approximately inversely with the beam radius. The beam radius coefficient is *h*_0_ = 100 pm·μm and the noise floor is *h_N_* = 20 pm. At a beam radius of 10 μm, the protein surface height resolution per pixel is 30 pm. Only the smooth spot, when scanned on the inner regions away from edges, deviates from this simple scaling. At a beam radius of 1 μm, the pixel height resolution is approximately 50 pm, corresponding to approximately 800 antibody molecules within the focal area of the laser beam.

In addition to studying the dependence of surface height resolution as a function of beam radius, the dependence on the radial pitch was also studied. The experimental results are shown in Figure 6b for a beam radius of 10 μm with 16 averages on the oscilloscope. The noise floor is again equal to 20 pm per pixel. The minimum resolvable height increases approximately quadratically with pitch. Interestingly, the smooth spot displayed a stronger dependence on pitch than the rough spot. However, the edges of the printed antibody spot were included in both cases, which likely dominates the uncertainty in successive measurements. For a beam radius of 10 μm and a pitch also equal to 10 μm, the surface height resolution is approximately 60 pm per pixel. 

## 5. Materials and Methods 

### 5.1 Microarray Fabrication

The substrates we used for the experiments were 100 mm diameter silicon wafers with a 120 nm thick thermal silicon dioxide layer. The reflectance change caused by protein molecules is optimized at a wavelength of 630 nm for this oxide thickness. Protein causes positive intensity shifts under these conditions, making it easy to distinguish protein signal from scattering loss caused by salt or dust particles. The oxide surface is chemically activated with two different surface chemistries (diisocyanate and butyraldehyde). The silicon wafer was separated into 96 independent wells by hydrophobic barriers on a BioCD [2], enabling incubation with different sample solutions without mixing. 

Proteins were prepared in a print buffer of pH 8.0 phosphate buffered saline (PBS) with 0.003% pluronic surfactants (BASF). The protein solutions were then printed in spot arrays by a piezoelectric printer (Scienion Inc., Monmouth Nunction, NJ, USA, distributed by BioDot) with 300 pL of protein solution per spot, resulting in approximately 100 μm diameter protein spots. Four protein spots were printed in a 2 × 2 unit cell pattern, with a total of 8 × 8 spots in a well. The printed proteins were rabbit IgG, polyclonal goat anti-rabbit IgG, chicken IgY and protein A/G. All proteins were from Sigma, and were prepared at a concentration of 100 μg/mL in the print buffer. The discs were printed with the protein solutions and incubated in a high humidity chamber at 37° and 70%–80% humidity. Post-print processing differed with different surface chemistries, and is described with each surface chemistry below.

### 5.2 Surface Chemistry

For the diisocyanate coating, the discs were plasma cleaned, followed by deposition of the hydrophobic barrier to separate the disc into independent wells. The surface was functionalized by vapor-phase deposition of aminopropaldimethyl ethoxy silane (APDMES) (Aldrich, Milwaukee, WI, USA) that covalently binds protein through a diisocyanate cross-linker.

For the diisocyanate discs, the post-print incubation lasted 1 h, followed by gas-phase blocking by ethanolamine for 45 min. The discs were placed in a vacuum chamber together with liquid-state ethanolamine, which evaporates in vacuum and reacts with the remaining functional diisocyanate surface. Because this reaction is reversible, ethanolamine only provides temporary blocking of the diisocyanate surface. The discs were washed on a spinner for 2 min with pH 7.4 PBS solution with 0.05% Tween 20 (PBST), followed by a 2-min wash with de-ionized (DI) water. The discs were spun dry at 3000 rpm on the spinner, and blocked for 30 min in a 0.05% casein solution prepared in PBST. Casein replaces ethanolamine molecules and permanently blocks the functional surfaces. After the casein blocking, the discs were washed again on the spinner for 2 min with PBST and 2 min with DI water. Then the discs were put into a stabilizing solution of 2% trehalose (Sigma) for 10 min and spun dry to preserve the activity of the printed protein molecules for prolonged storage.

For the butyraldehyde surface chemistry, the discs were cleaned by an SC-1 process that heats the discs in a 10:1:1 dilute solution of DI water, 30% hydrogen peroxide and 30% ammonium hydroxide. The disc surface was silanized by gas-phase deposition of triethoxysilyl butyraldehyde (Gelest, Morrisville, NY, USA), after which the hydrophobic barrier was deposited onto the surface. The amino groups on the protein react with the carbonyl group of butyraldehyde to form a Schiff base. The surface is then reduced by a sodium cyanoborohydride (Aldrich) solution to stabilize the protein immobilization by cross-linking the antibody with multiple surface groups.

For the butyraldehyde discs, the post-print incubation lasted 3 h, followed by a 3 min wash with 50 mM citrate buffer at pH 6.0 with 0.05% Tween 20, and a one-min wash with DI water. After the spinner wash, the discs were submerged in PBS solution and washed on an orbital shaker for 15 min. The discs were then submerged into a 0.5 mg/mL sodium cyanoborohydride solution prepared in 2× PBS at pH 8.0 with 10% ethanol for 5 min to reduce the butyraldehyde surface. After reduction, the discs were washed with citrate buffer on an orbital shaker for 30 min. The discs were rinsed on the spinner for 2 min with a PBST wash followed by 1 min DI water wash and spun dry. The discs were then passivated with 0.05% casein solution prepared in PBST for 1 h. After passivation the discs were rinsed with PBST for 3 min followed by 1 min of DI water, then they were stabilized with 2% trehalose solution for 10 min and spun dry.

Protein A/G is a recombinant fusion protein that has the IgG binding domain of both protein A and protein G [21]. Both protein A and protein G bind specifically to the Fc region of the IgG molecules, and have been widely used to provide oriented immobilization of the antibody molecules [22,23]. Compared to the parent proteins that have limited binding to several species and subclasses of IgG, the chimeric protein A/G binds all sub-classes of mammalian IgG with high affinity. The multiple binding sites on a protein A/G molecule also make it relatively robust to retain most of its reactivity when immobilized onto a solid surface. For protein A/G immobilization of antibodies, protein A/G was first printed onto the substrate in spots, with both surface chemistries. When the protein A/G spots were subsequently incubated with a sample containing IgG, the molecules were captured onto the spots and immobilized. 

## 6. Conclusions 

The experimental results of the previous section represent the ideal metrology limits for detection of printed antibody spots on the in-line interferometric BioCD. The studies involved two-dimensional scans with rescans of the same spots in the absence of assay chemistry. As stated before, assay chemistry sets the practical limits on protein detection on the BioCD because of possible wash-off of printed protein as well as deposition of residues. However, the results in this paper focus entirely on the ideal metrology limits under the same scan conditions that are practiced in the binding assays, thereby setting the lowest possible bound on detected protein. Using the scan and rescan results, it is possible to set the minimum detectable binding limit for the BioCD.

The surface height sensitivity for antigen binding to of a printed antibody spot is given by:

(7) $$\Delta h_{spot}=\frac{\Delta h_{pix}}{\sqrt{N}}=\Delta h_{pix}\frac{r}{R}$$

for optimal scanning (pitch equal to beam radius), where r is the beam radius and *R* is the antibody spot radius. For a printed antibody spot of 50 μm radius scanned at 1 μm pitch with a 1 μm beam radius, $\Delta h_{spot}$ equals 1 pm. Assuming a density for the bound antibody material equal to the density of water, this metrology limit equals 10 fg of bound material per spot. If the target antigen is a 150 kDa macromolecule, this equals 30 × 10^6^ bound molecules per spot. The speed of rotation does not play a direct role in the sensitivity of the BioCD, but slower speeds would require longer read times to measure the same number of repetitions.

It is important to point out that this metrology limit is an extreme lower limit. Under practical assay conditions, including incubation and wash off, the role of residues bound to the surface can raise the assay limit from 1 pm to tens of picometers (with an assay limit of detection of approximately 1 ng/mL). However, the work presented here does set the possible range for binding experiments that may not use traditional wet chemistry. For instance, electrospray deposition [24] is a method of producing an aerosol of macromolecules that can be deposited on solid surfaces. If a BioCD disk is spinning and actively reading during electrospray deposition, this would be a deposition condition that could approach the metrology limit described in this paper. Indeed, in such a deposition experiment, 2D scanning would not be necessary, utilizing single-track scanning. Assuming the 10 pm surface height resolution per pixel for single-track scanning during electrospray deposition for a beam radius of 1 μm, this represents a mass detection sensitivity per spot of 140 attograms corresponding to half a million detected antigen molecules per spot.

In conclusion, the protein surface height sensitivity of the in-line phase-sensitive BioCD is nearly shot-noise limited (within a factor of 5) for single-track scanning that has no repositioning error except for small shifts in angular speed governed by the spinning motor. In 2D scanning, the surface height sensitivity is a function of both the beam radius as well as the radial scan pitch. The metrology limit for 2D scanning of a printed antibody spot is approximately 1 pm per 100 μm diameter spot.

## Abbreviations

The following abbreviations are used in this manuscript:

BioCD: Biological compact disc
APDMES: Aminopropaldimethyl ethoxy silane
PBS: Phosphate buffered saline
SDI: Spinning disk interferometry
